# Refined Candidate Region for F4ab/ac Enterotoxigenic *Escherichia coli* Susceptibility Situated Proximal to *MUC13* in Pigs

**DOI:** 10.1371/journal.pone.0105013

**Published:** 2014-08-19

**Authors:** Tiphanie Goetstouwers, Mario Van Poucke, Wouter Coppieters, Van Ut Nguyen, Vesna Melkebeek, Annelies Coddens, Katleen Van Steendam, Dieter Deforce, Eric Cox, Luc J. Peelman

**Affiliations:** 1 Laboratory of Animal Genetics, Faculty of Veterinary Medicine, Ghent University, Belgium; 2 Unit of Animal Genomics, GIGA-R and Faculty of Veterinary Medicine, University of Liège (B34), Liège (Sart Tilman), Belgium; 3 Laboratory of Immunology, Faculty of Veterinary Medicine, Ghent University, Merelbeke, Belgium; 4 Laboratory of Pharmaceutical Biotechnology, Faculty of Pharmaceutical Sciences, Ghent University, Ghent, Belgium; Cornell University, United States of America

## Abstract

F4 enterotoxigenic *Escherichia coli* (F4 ETEC) are an important cause of diarrhea in neonatal and newly-weaned pigs. Based on the predicted differential O-glycosylation patterns of the 2 MUC13 variants (MUC13A and MUC13B) in F4ac ETEC susceptible and F4ac ETEC resistant pigs, the *MUC13* gene was recently proposed as the causal gene for F4ac ETEC susceptibility. Because the absence of MUC13 on Western blot from brush border membrane vesicles of F4ab/acR^+^ pigs and the absence of F4ac attachment to immunoprecipitated MUC13 could not support this hypothesis, a new GWAS study was performed using 52 non-adhesive and 68 strong adhesive pigs for F4ab/ac ETEC originating from 5 Belgian farms. A refined candidate region (chr13: 144,810,100–144,993,222) for F4ab/ac ETEC susceptibility was identified with *MUC13* adjacent to the distal part of the region. This candidate region lacks annotated genes and contains a sequence gap based on the sequence of the porcine GenomeBuild 10.2. We hypothesize that a porcine orphan gene or trans-acting element present in the identified candidate region has an effect on the glycosylation of F4 binding proteins and therefore determines the F4ab/ac ETEC susceptibility in pigs.

## Introduction

*F4 enterotoxigenic Escherichia coli* (F4 ETEC) diarrheal disease in neonatal and newly-weaned pigs is an economically important genetic disease inherited in an autosomal dominant pattern [1]–[3]. F4 ETEC possess F4 fimbriae acting as ligands for specific carbohydrate receptors on the epithelial surface of the small intestine. This interaction results in colonization of the small intestine and in production of enterotoxins inducing a secretory diarrhea in young pigs [2]. F4 fimbriae exist in 3 antigenic variants: F4ab, F4ac and F4ad, of which F4ac is the most prevalent, except in central China where F4ad is the most prevalent fimbrial variant [2], . In the search for the causal mutation for F4ab/ac ETEC susceptibility, several linkage studies mapped the causal mutation(s) on chromosome 13 [5]–[7]. Some studies suggest that one locus is controlling both F4ab and F4ac ETEC susceptibility [8]–[10], while others suggest 2 linked but distinct loci [11]–[16]. The g.8227G>C polymorphism in *MUC4* [Genbank:DQ848681] was found to be strongly associated with F4ab/ac ETEC susceptibility with the dominant G allele representing F4ab/ac ETEC susceptibility and the recessive C allele representing F4ab/ac ETEC resistance. A genotyping test was proposed for distinguishing F4ab/ac ETEC susceptible and resistant pigs [17]. However, Rasschaert *et al.* [18] could not confirm this when comparing with *in vitro* adhesion to villi. Recently, Ren *et al.* [6] suggested that 2 MUC13 variants (MUC13A and MUC13B) are responsible for F4ac ETEC susceptibility of Duroc x Erhualian, Chinese conventional Sutai and Duroc x Landrace x Large White hybrids populations, due to a (predicted) different O-glycosylation pattern between these 2 proteins. In this study, we investigated if *MUC13* is responsible for F4ab/ac ETEC susceptibility and we performed a GWAS study to identify a refined candidate region.

## Materials and Methods

### Sample collection

Experimental and animal management procedures were approved by the animal care and ethics committee of the Faculty of Veterinary Medicine, University of Ghent (EC2010/042). Pigs originating from 5 Belgian farms were euthanized at 6–18 weeks of age. The breeds of these pigs were Large White, Belgian Landrace, Large White x Belgian Landrace crossbreds, Large White x Piétrain crossbreds, and crossbreds of multiple breeds. Before euthanasia, blood samples were collected in EDTA blood tubes for DNA analysis and stored at −20°C. After euthanasia, two-meter mid-jejunum samples were washed 2 times with Krebs-Henseleit buffer (0.12 M NaCl, 0.014 M KCl, 0.001 M KH2PO4, 0.025 M NaHCO3, pH 7.4) and once with Krebs-Henseleit buffer containing 1% (v/v) formaldehyde at 4°C. After washing, the villi were scraped from the mucosa of a 20 cm segment and stored as mentioned in Van den Broeck *et al.* [19]. These villi were used for the *in vitro* villous adhesion assay. The rest of the mid-jejunal sample was used to isolate brush border membrane vesicles (BBMV) as described in Nguyen *et al.* [20].

### F4ab/acR phenotyping based on the *in vitro* villous adhesion assay

All pigs were phenotyped for the presence of the F4ab/ac receptor (F4ab/acR) using the *in vitro* villous adhesion assay with 4×10^8^ F4ac *E. coli* (strain GIS26, serotype O149:K91, F4ac^+^) or F4ab *E. coli* (strain G7, serotype O8:K87, F4ab^+^) and an average of 50 villi in a volume of 0.5 ml PBS with 1% (W/V) D-mannose [19]. Adhesion of more than 30 bacteria per 250 µm villous brush border length was noted as strong adhesive for F4ab/ac ETEC and less than 5 bacteria per 250 µm brush border length was noted as non-adhesive for F4ab/ac ETEC [21]. A total of 120 pigs from 43 different litters were included in the GWAS study based on the 2 F4ab/acR phenotypes: non-adhesive (F4R^−^; n = 52) and strong adhesive (F4R^+^; n = 68). The two F4ab/acR phenotypes were present in 23 litters, in 10 litters only the non-adhesive F4ab/acR phenotype and in 10 other litters only the strong adhesive F4ab/acR phenotype was present. Nine boars in this study had multiple litters (Table S1).

Pigs that showed a weak adhesion towards F4ab/ac ETEC were excluded.

### *MUC4* TaqMan assay and the Indel *MUC13* marker test

DNA isolation of the blood samples for the *MUC4* TaqMan assay and the Indel *MUC13* marker was performed as described by Van Poucke *et al.* [22].

The *MUC4* TaqMan assay was carried out as described by Nguyen *et al.* [20] and is based on the g.8227G>C mutation of *MUC4* [Genbank:DQ848681] associated with F4ab/ac ETEC susceptibility [17]. The protocol for the *MUC4* TaqMan assay is provided as supporting information; see Protocol S1.

The Indel *MUC13* marker test is based on an Indel of 68 bp in intron 2 of *MUC13* [GenBank:NC_010455.4; 144,993,222–144,993,289] differentiating *MUC13A* (the longer sequence) and *MUC13B* (the shortened sequence) and was performed as described by Ren *et al.* [6].

### Expression of the MUC13 glycoprotein and F4ac binding proteins in BBMVs

In a recent study Nguyen *et al.* [20] demonstrated that two high molecular weight (MW) glycoproteins (>250 kD), which bind F4ab and F4ac fimbriae, are consistently present in pigs with the *MUC4* homozygote or heterozygote susceptible genotype that show an immune response upon oral immunization with F4ac fimbriae. Here, these high MW glycoproteins were purified from F4ab/acR^+^ BBMVs group I [20] using anion exchange (Pierce strong anion exchange spin column, Thermo Scientific) followed by gel filtration (HiPrep 16/60 Sephacryl S-200, GE Healthcare). Eluates were tested by one-dimensional immunoblotting for the presence of the high MW glycoproteins with biotinylated F4ac fimbriae as described in Nguyen *et al.* [20].

A peptide (FPKIKVDISRGGQP) selected from the C-terminus of the porcine MUC13B sequence was synthesized and conjugated to keyhole limpet hemocyanin (KLH) (Genscript, Piscataway, USA) [6]. The peptide conjugate emulsified in incomplete Freund's adjuvant was used to immunize rabbits. Immunoglobulin G was purified using a Hitrap™ Protein G HP column (GE Healthcare) [23]. These antibodies dissolved in PBS were used to stain immunoblots of BBMVs from F4ab/acR^+^ pigs separated by SDS-PAGE under reducing and non-reducing conditions [20]. In parallel, the binding pattern of biotinylated F4ac fimbriae to these blots was determined.

Additionally, MUC13 was precipitated from BBMVs by incubating 1 mg of the vesicles with 50 µg/ml anti-MUC13 antibodies for 1 h at 4°C followed by 50 µl protein A conjugated sepharose (Protein A Sepharose ™ CL-4B, GE Healthcare) for 1 h at 4°C. Precipitated MUC13 was eluted from the sepharose by boiling it for 10 min at 95°C in 100 µl SDS-PAGE reducing loading buffer. Subsequently SDS-PAGE and immunoblotting with F4ac fimbriae were performed to determine the F4ac binding pattern [20].

### Genome-wide association study

DNA isolation of the blood samples for the genome-wide association study was performed as described by Dupuis *et al.* [24]. A total of 120 F4ab/acR phenotyped pigs were genotyped using the Porcine SNP60 BeadChip (Illumina) containing 62,163 SNPs, according to the manufacturer's protocol. The position of the SNPs was based on the current pig genome assembly (Sscrofa10.2). Quality scores were analyzed from allele cluster definitions for each SNP as determined by the Illumina GenomeStudio Genotyping Module version 1.0 (Illumina). All genotype calls were extracted from the raw data with a minimum gencall score threshold of 0.2. An additional quality control was performed using PLINK [25]. SNPs were filtered with a call rate >95%, GenTrain Score <0.7 and minor allele frequency (MAF) ≥0.01. A total of 6874 (11%) SNPs were excluded from further analysis. Two SNPs, namely the *MUC4* SNP and the *MUC13* SNP, were added in our association analysis based on the g.8227G>C mutation of *MUC4* [Genbank:DQ848681] associated with F4ab/ac ETEC susceptibility and based on the Indel of 68 bp in intron 2 of *MUC13* [GenBank:NC_010455.4; 144,993,222–144,993,289] [6], [17]. Pigs were genotyped for the *MUC4* SNP by performing the *MUC4* TaqMan assay as described by Nguyen *et al.* [20].

No samples were excluded due to frequency of missing genotypes >5%. Population stratification based on pair-wise identity-by-state (IBS) distances was also quantified using the PLINK software [25]. Multidimensional scaling (MDS) identified 5 clusters corresponding to breed with no genotypic outliers and the 2 F4ab/acR phenotypes were present in each cluster. These 5 clusters were used as stratification criteria for following association analysis.

Association between the SNPs and the F4ab/ac receptor positive (F4ab/acR^+^) status was assessed using the 2×2xK Cochran-Mantel-Haenszel (CMH) test for 5 clusters.

**Figure 1:**
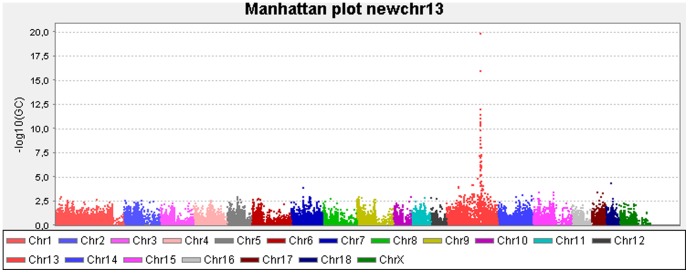
Manhattan plot obtained from the *P*-values for F4ab/ac ETEC susceptibility in 120 pigs. SNPs are plotted on the X-axis ordered by chromosomal position. Genome-wide -log(10) *P*-values adjusted to genomic control are plotted on the Y-axis.

The Bonferroni correction and the Max (T) permutation procedure (10,000 permutations) within the breeds were used to correct for experiment-wise error rate (Table S2) [26]. Manhattan plots of the results were generated using Haploview (Figure 1) [27].

## Results

### *MUC4* TaqMan assay and the Indel *MUC13* marker test

Using the *MUC4* TaqMan assay 17 pigs (25%) were genotyped as homozygous susceptible (SS), 41 pigs (60.3%) as heterozygous susceptible (SR) and 10 pigs (14.7%) as homozygous resistant (RR) in the F4ab/ac ETEC strong adhesive group (n = 68). In the F4ab/ac ETEC non-adhesive group (n = 52), 4 SR pigs (7.7%) and 48 RR pigs (92.3%) were present (Table S1).

The Indel *MUC13* marker test showed that 50 pigs (73.5%) were homozygous for the *MUC13B* allele (BB) and 18 pigs (26.5%) had a *MUC13A* as well as a *MUC13B* allele (AB) in the F4ab/ac ETEC strong adhesive group (n = 68). In the F4ab/ac ETEC non-adhesive group (n = 52), 8 pigs (15.4%) were BB, 27 pigs (51.9%) were AB and 17 pigs (32.7%) were homozygous for the *MUC13A* allele (AA) (Table S1).

### F4ac fimbriae do not bind to MUC13 glycoprotein of BBMVs

**Figure 2:**
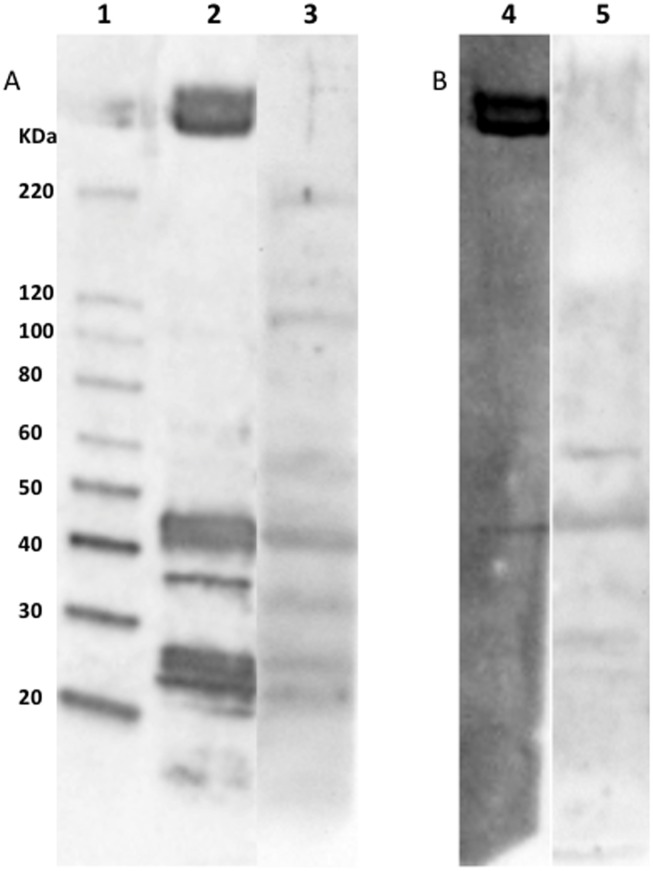
Immunoblotting of F4ab/acR^+^ BBMVs with F4ac fimbriae (lane 2 and 4) or anti-MUC13 antibodies (lane 3 and 5). Proteins were separated under reducing (A) and non-reducing (B) conditions. F4ac fimbriae bound to the F4-specific high molecular weight glycoproteins (only present in F4R^+^ pigs) and several non-specific F4-binding bands <50 kDa (present in F4R^+^ and F4R^−^ pigs) [20]. Anti-MUC13 antibodies recognized BBMV protein bands of 55, 47, 34 and <25 kDa under reducing and non-reducing conditions, but bands of 200, 110 kDa only under reducing conditions. Lane 1  =  protein standards.

Separating BBMVs by SDS-PAGE under reducing (Figure 2A) and non-reducing conditions (Figure 2B) followed by immunoblotting with biotinylated F4ac fimbriae (lanes 2 and 4) or anti-MUC13 antibodies (lanes 3 and 5) revealed bands with a similar molecular weight (47, 34 and <25 KDa). These bands are not F4-specific bands since they are presented in both F4R^+^ and F4R^−^ BBMV blots [20]. However, it is also clearly demonstrated that the anti-MUC13 antibodies did not bind to the high MW glycoproteins which are specifically recognized by F4ac fimbriae being only present in F4R^+^ and not in F4R^−^ BBMV blots.

**Figure 3:**
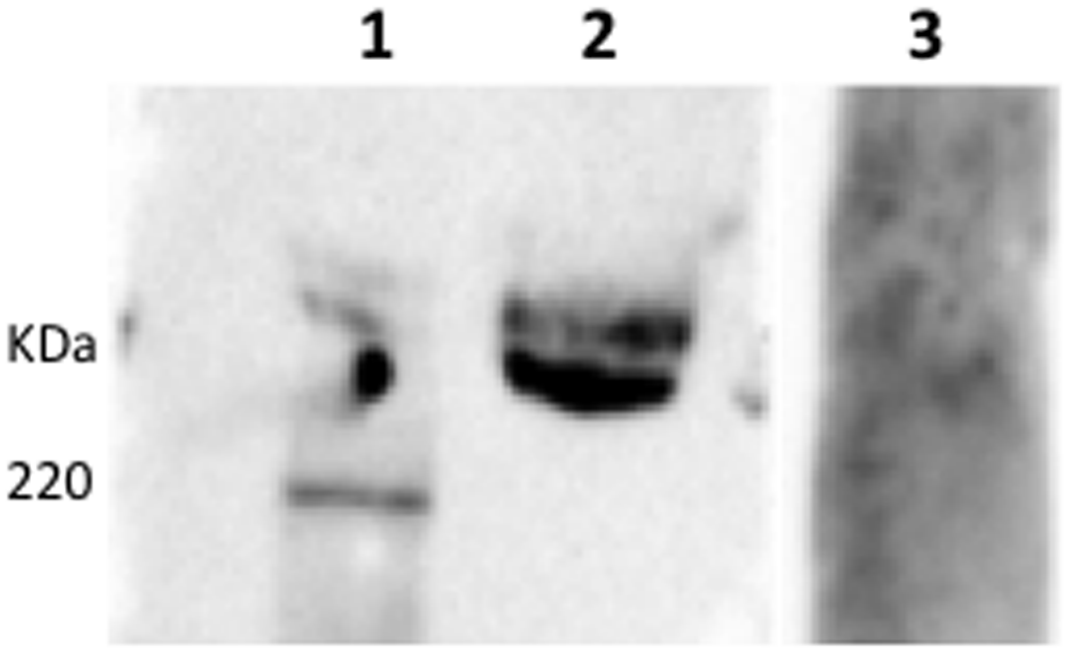
Absence of detection of intestinal MUC13 (lane 3) by anti-mucin 13 antibodies in the purified high molecular weight (MW) fraction of F4ab/acR^+^ BBMVs. Purification occurred by anion exchange chromatography followed by gel filtration chromatography. Strong binding of F4ac fimbriae to the high MW glycoproteins can be seen in lane 2. Lane 1: protein standard.

**Figure 4:**
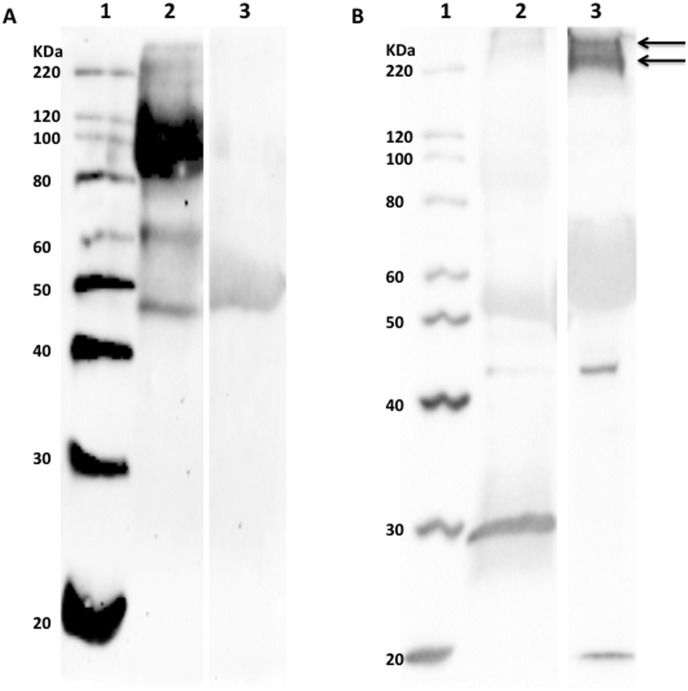
Absence of F4ac fimbriae binding to immunoprecipitated intestinal MUC13. Intestinal MUC13 was purified from 1 mg F4ab/acR^+^ brush border membrane vesicles (BBMVs) by immunoprecipitation with anti-MUC13 antibodies and protein A sepharose. The eluate (lane 2) and the non-precipitated fraction (lane 3) were immunoblotted with anti-MUC13 antibodies (A) or with F4ac fimbriae (B). Immunoprecipitation enriched a protein with a band of 110 kDa but this was not recognized by F4ac fimbriae. The F4-specific high MW glycoproteins were found in the non-precipitated fraction. Lane 1  =  protein standards. Arrows: position of the high MW glycoproteins.

Purifying and enriching the high MW glycoproteins by a combination of anion exchange chromatography and gel filtration did not change this (Figure 3 lane 3). Immunoprecipitation of MUC13 with the anti-MUC13 antibodies enriched a protein with MW of 110 kDa (Figure 4A lane 2) but this was not recognized by F4ac fimbriae (Figure 4 A and B lane 2). Furthermore, not the immunoprecipitated fraction, but the non-immunoprecipitated fraction contained the F4-specific high MW glycoproteins (Figure 4B lane 3). This result excludes MUC13 as one of the F4-specific high MW glycoproteins.

### Association Analysis

**Figure 5:**
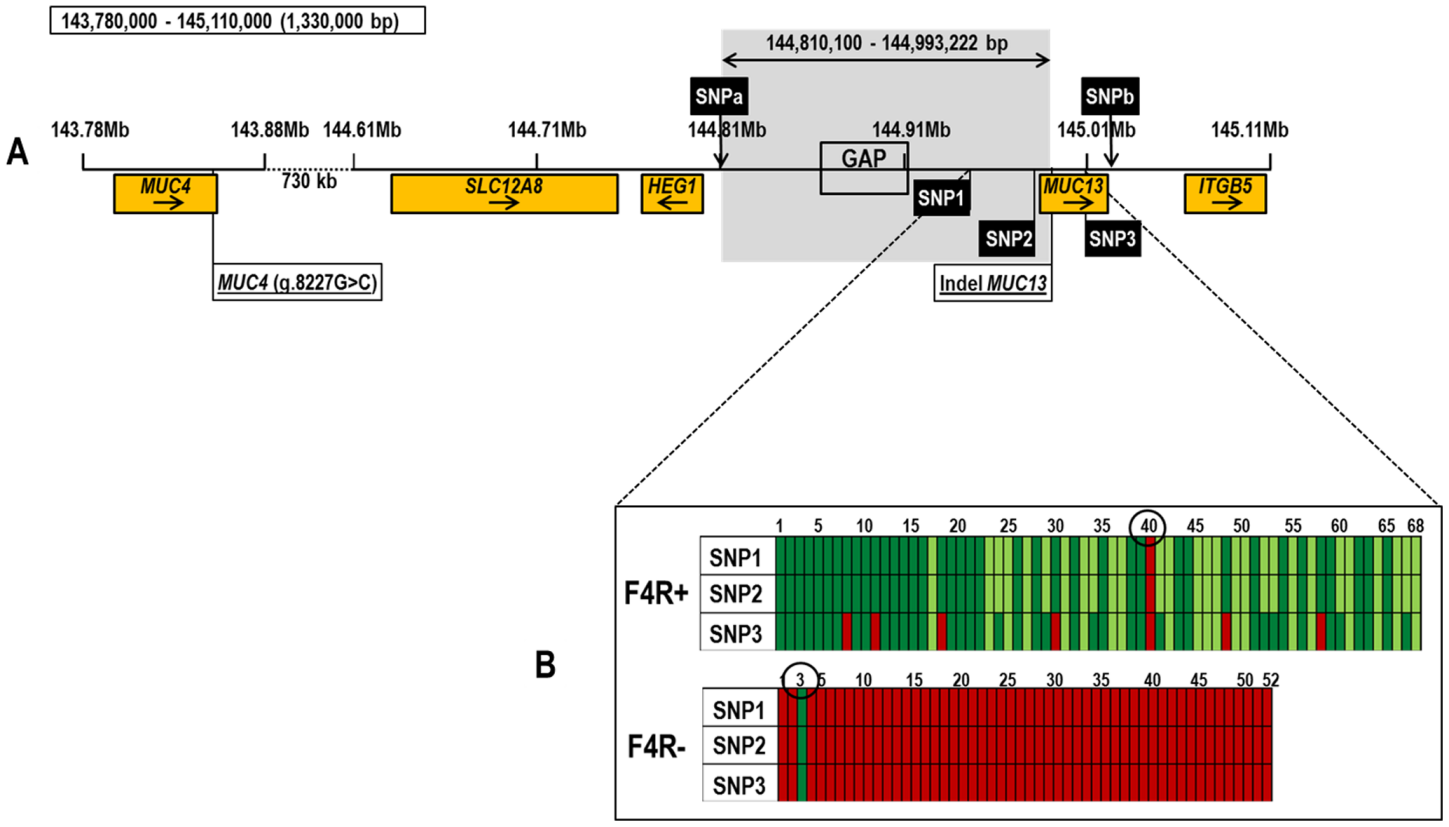
Schematic representation showing the identified candidate region (chr13: 144,810,100-144,993,222) of F4ab/ac ETEC susceptibility between MARC0002946 (SNPa) and Indel *MUC13* marker on chromosome 13 (chr13: 143,780,000-145,110,000). (**A**) SNP1 (ASGA0089965), SNP2 (ASGA0091537) and SNP3 (ALGA0106330) are the most significant SNPs in the association study. MARC0002946 (SNPa) and ALGA0106230 (SNPb) are not associated with F4ab/ac ETEC susceptibility. The orange boxes represent all the annotated genes in the 1.33 Mb region of chromosome 13. The gray box represent the candidate region where no annotated genes were found during the *in silico* comparative mapping. (**B**) Schematic representation showing the genotypes of SNP1 (ASGA0089965), SNP2 (ASGA0091537) and SNP3 (ALGA0106330) of 68 strong adhesive (F4R^+^) and 52 non-adhesive (F4R^−^) for F4ab/ac ETEC. For SNP1 and SNP3, the dark green boxes represent CC genotype, light green boxes represent CT genotype and red boxes represent TT genotype. For SNP2, the dark green boxes represent TT genotype, light green boxes represent CT genotype and red boxes represent CC genotype. Pig 40 (F4aR^+^) and pig 3 (F4R^−^) show different genotypes for the markers than expected.

The dataset presented in this article has been submitted to the Gene Expression Omnibus (http://www.ncbi.nlm.nih.gov/geo/) under accession number GSE57981. The results of the SNP association significance are visualized in a Manhattan plot of genome-wide-log(10) *P*-values adjusted to genomic control (Figure 1). Outliers displaying the highest association *P*-values of the SNPs are only visible on chromosome 13. After controlling for multiple testing, 42 SNPs reached genome-wide significance (<0.05): 40 SNPs are located within an interval of 5.7 Mb on SSC13 and the position of 2 SNPs (ALGA0122702 and M1GA0027009) are unknown in the GenomeBuild 10.2 (Table S2). The 3 most significant SNPs are located in a region of 63,063 bp on chromosome 13 close to *MUC13*. SNP1 (ASGA0089965) and SNP2 (ASGA0091537) have the same *P*-value and are completely linked, only 1 strong adhesive F4Rab/ac pig (1/68) and 1 non-adhesive F4ab/ac pig (1/52) have a different genotype for these SNPs than expected. For SNP3 (ALGA0106330), 7 strong adhesive F4ab/ac pigs (7/68) and 1 non-adhesive F4ab/ac pig (1/52) have a different genotype than expected (Figure 5B). A region of 213,267 bp (chr13: 144,810,100–145,023,367) was considered as the candidate region for F4ab/ac ETEC susceptibility. The borders of this region are 2 unassociated SNPs, namely MARC0002946 (SNPa) and ALGA0106230 (SNPb) (Figure 5A).

## Discussion

The results of the *MUC4* TaqMan assay confirm that the g.8227G>C mutation of *MUC4* is associated with F4ab/ac ETEC susceptibility [17]. Nevertheless, the genotypes, especially the RR genotype, were not completely consistent with the results of the *in vitro* villous adhesion assay. Ten of the RR pigs (14.7%) showed adhesion towards F4ab/ac ETEC and 4 SR pigs (7.7%) showed no adhesion. This result confirms earlier findings that the g.8227G>C mutation is a marker but not the actual causative mutation [18], [28].

Recently, *MUC13* was suggested as the causal gene for F4ac ETEC susceptibility [6]. The MUC13 glycoprotein consists of an α and β subunit interconnected by covalent links which can be cleaved by proteases [29]. The β subunit refers to the C-terminal subunit containing the cytoplasmic tail and forms as homodimer. The α subunit carries weighty O-glycosylations, which are thought to be responsible for the F4 ETEC binding. Unlike MUC13A that lacks an O-glycosylation site, MUC13B is predicted to be heavily O-glycosylated and therefore would contain the binding site for F4ac ETEC [6]. This hypothesis suggests that the *MUC13B* allele (present in F4ac ETEC susceptible pigs) is dominant over the *MUC13A* allele (present in the F4ac ETEC resistant pigs). By performing the Indel *MUC13* marker test in 120 F4ab/acR phenotyped pigs, the presence of the *MUC13B* allele was identified in 35 F4ab/ac ETEC non-adhesive pigs (67.3%), rejecting the hypothesis (Table S1).

The predicted mass of the entire MUC13B protein is about 44 kDa based on the MUC13B sequence [6], but the expected mass on SDS gel should be much higher due to massive glycosylation and a gap in the domain rich in the amino acids Proline, Threonine and Serine (PTS domain) of the DNA sequence.

The peptide used to immunize rabbits was selected from the MUC13B sequence described by Ren *et al.* [6]. It is located in the cytoplasmic tail of the β subunit and is predicted not to contain glycosylation sites based on DictyOGlyc and NetNGlyc [6]. Consequently, in theory, the produced antibodies should bind to the cytoplasmic parts of MUC13 without interference of glycans at the glycosylated sites. Binding of the MUC13 antibodies to BBMV proteins was tested under non-reducing and reducing conditions so that both intact MUC13 and the individual monomers with digestion products should be observed. As expected, more bands were recognized in the reducing condition. Nguyen *et al.* [20] demonstrated that the three F4 variants bound specifically the two high molecular weight (MW) glycoproteins (>250 kD), while the other bands (130, 110, 75, 64, 50, 47, 43, 40, 34, 32, and <25 kDa) were also recognized by F4 fimbriae but not specifically since they were present in both F4R^+^ and F4R^−^ BBMV blots [20]. In the present study, anti-MUC13 antibodies and F4ac fimbriae also bound the 47, 34, and <25 KDa bands, which are not F4-specific. However, in none of the conditions MUC13 antibodies recognized the F4-specific high MW glycoproteins. Furthermore, the immunoprecipitated MUC13 was not recognized by F4ac fimbriae. These results exclude MUC13 as one of the F4-binding high molecular weight glycoproteins and F4 fimbriae do not bind specifically to MUC13. Also by using NetOGlyc 4.0 which is a mucin-type (GalNAc) glycosylation predictor trained on mammalian protein sequences [30], it was predicted that MUC13A (AEO00194.1) as well as MUC13B (AEO00200.1) are O-glycosylated.

Based on our results, we could conclude that the 2 MUC13 variants are not responsible for susceptibility towards F4ab/ac ETEC.

Our GWAS study demonstrates a strong association between 3 SNPs (ASGA0089965, ASGA0091537, ALGA0106330) and the F4ab/acR locus. In 118 pigs (98.33%), 2 markers, namely SNP1 (ASGA0089965) and SNP2 (ASGA0091537), were in complete linkage disequilibrium (LD) with the F4ab/acR locus. For 2 pigs (1 strong adhesive F4ab/ac pig and 1 non-adhesive F4ab/ac pig), the F4ab/acR phenotype showed the opposite genotype for these markers (Figure 5B). It is possible that the causal mutation is located proximal of SNP1 or that the F4ab/acR phenotype is more complex and regulated by multiple factors.

Our results confirm that *MUC4* and *MUC13* are not completely associated with F4ab/ac ETEC susceptibility. In our association analysis, the *MUC4* marker (g.8227G>C mutation) and the Indel *MUC13* marker [GenBank:NC_010455.4; 144,993,222-144,993,289] were included. The *MUC4* marker showed a lower significant *P*-value than 4 markers located more distal from the g.8227G>C mutation (Table S2; Figure 5A). The low significant *P*-value of the Indel *MUC13* marker (Table S2; Figure 5A) confirmed our previous results that *MUC13* is not the causal gene for F4ab/ac ETEC susceptibility. Four markers of the Porcine SNP60 BeadChip (ALGA0072075 [GenBank:NC_010455.4; 144832256], ALGA0106330 (SNP3), DIAS0000584 [GenBank:NC_010455.4; 145414240] and MARC0006918 [unknown position]), and 2 additional markers (*MUC13*-226 [GenBank:NC_010455.4; 145,010,437] and *MUC13*-813 [GenBank:NC_010455.4; 145,016,914]) were in complete LD with the F4ab/acR locus in a Swiss experimental herd [31]. Except for one sow and some of her offspring, markers ALGA0106330 (SNP 3), *MUC13*-226 and *MUC13*-813 were not in LD with the F4ab/acR locus [32]. In our study, the markers ALGA0072075, ALGA0106330 (SNP 3), and DIAS0000584 all had statistically significant *P*-values (Table S2), but showed a weaker F4ab/acR association than SNP1 and SNP2. The unmapped marker MARC0006918 was not associated with the F4ab/acR locus in our study. Because *MUC13*-226 and *MUC13*-813 are positioned distal to the Indel *MUC13* marker, we refined the candidate region to 183,122 bp (chr13: 144,810,100-144,993,222; Figure 5A).

Also, our 3 most significant SNPs were significant in another F4ab/ac ETEC susceptibility study [5], but other more proximal located SNPs (MARC0012378; M1GA0017682; ALGA0072075) in this study showed a higher significant *P*-value.

This result could be due to the fact that they included the F4ab/acR phenotype weak adhesive as non-adhesive pigs.

The refined candidate region of 183,122 bp (chr13: 144,810,100-144,993,222) on chromosome 13 is almost in complete linkage with the F4ab/acR phenotype. No porcine annotated genes are present in our candidate region based on the available genome sequence of the porcine GenomeBuild 10.2 (Figure 5A). Comparison of the candidate region with orthologous regions of human, mouse and rat genomes (GenBank:NC_000003.11 (human); NC_0000826 (mouse); NC_005110.3 (rat)) using BLAST analysis was performed to identify non-annotated porcine genes as well as identifying the gap sequence (between NW_003611795.1 and NW_003617796.1) present in the candidate region [33]. Performing the interspecies comparison, no new non-annotated porcine genes were identified and we were unable to identify the gap sequence. The presence of a porcine orphan gene in the candidate region cannot be ruled out. Fang *et al.* [39] identified 240 orphan genes with no counterpart in any other organism (human, horse, dog, cat, cattle, rat and mouse) during analysis of the genome sequence of the Wuzhishan miniature pig. Also, the candidate region could contain a trans-acting element interacting with a distant gene influencing F4 ETEC adherence in pigs.

Based on previous F4 ETEC binding studies, it was concluded that the carbohydrate moiety of glycoconjugates appears to be necessary for establishing adhesion with the F4 adhesin [34]–[38]. We propose that a porcine orphan gene or a trans-acting element present in the candidate region (chr13: 144,810,100-144,993,222) has an effect on the glycosylation of F4 binding proteins and therefore determines the F4 ETEC susceptibility in pigs.

## Supporting Information

Table S1 Information about the pigs used in the *MUC4* TaqMan assay, the Indel *MUC13* marker test, and the GWAS study. ^a^Breeds are defined as follows: ‘LW’ denotes Large White, ‘BL’ denotes Belgian Landrace, ‘LW x BL’ denotes Large White x Belgian Landrace crossbreds, ‘LW x P’ denotes Large White x Piétrain crossbreds, ‘hybrid’ denotes crossbreds of multiple breeds; ^b^ *MUC4* genotypes are defined as follows: ‘SS’ denotes homozygous susceptible (S allele is corresponding with the G allele), ‘SR’ denotes heterozygous susceptible (R allele is corresponding with the C allele), ‘RR’ denotes homozygous resistant; ^c^ *MUC13* alleles are defined as follows: ‘AA’ denotes homozygous for the *MUC13A* allele, ‘AB’ denotes both *MUC13A* and *MUC13B* alleles are present, ‘BB’ denotes homozygous for the *MUC13B* allele.
(DOCX)

Table S2 Significant SNPs for F4ab/ac ETEC susceptibility. ^a^Derived from porcine GenomeBuild 10.2; ^b^The nearest annotated porcine gene to the significant SNP based on the porcine GenomeBuild 10.2. Numbers in parentheses indicate distance in base pairs (bp); ^c^Genomic control corrected significance value. This is based on a simple estimation of the inflation factor based on median chi-square statistic (genomic inflation factor λ is 1.26636) [25].
(DOCX)

Protocol S1 **Protocol of the** ***MUC4*** **TaqMan assay using the iCycler iQ Real-Time PCR Detection System Software version 3.0a (Bio-Rad Laboratories, USA).** (**A**) Oligonucleotide sequences of primers and probes with their specifications (*MUC4*: DQ848681). Fluorescent labels and quenchers are in *italic*; SNPs are underlined. (**B**) PCR-mix (10 µl) used in the *MUC4* TaqMan assay by Real-Time PCR. (**C**) PCR program used in the *MUC4* TaqMan assay by Real-Time PCR.
(DOCX)
