# Supplementary material for: Refined Candidate Region for F4ab/ac Enterotoxigenic Escherichia coli Susceptibility Situated Proximal to MUC13 in Pigs
Source: PLoS One. 2014 Aug 19;9(8):e105013. doi: 10.1371/journal.pone.0105013 (PMC4138166; doi:10.1371/journal.pone.0105013)
Supplement: Table S1 — Information about the pigs used in the MUC4 TaqMan assay, the Indel MUC13 marker test, and the GWAS study. aBreeds are defined as follows: ‘LW’ denotes Large White, ‘BL’ denotes Belgian Landrace, ‘LW x BL’ denotes Large White x Belgian Landrace crossbreds, ‘LW x P’ denotes Large White x Piétrain crossbreds, ‘hybrid’ denotes crossbreds of multiple breeds; b MUC4 genotypes are defined as follows: ‘SS’ denotes homozygous susceptible (S allele is corresponding with the G allele), ‘SR’ denotes heterozygous susceptible (R allele is corresponding with the C allele), ‘RR’ denotes homozygous resistant; c MUC13 alleles are defined as follows: ‘AA’ denotes homozygous for the MUC13A allele, ‘AB’ denotes both MUC13A and MUC13B alleles are present, ‘BB’ denotes homozygous for the MUC13B allele. (DOCX) [file pone.0105013.s001.docx]

Table S1.

| **F4R^+^ pigs** | **Litter** | **Sow** | **Boar** | **Breed**^a^ | ***MUC4* (g.8227G>C)**^b^ | **Indel *MUC13***^c^ | **F4R^-^pigs** | **Litter** | **Sow** | **Boar** | **Breed**^a^ | ***MUC4* (g.8227G>C)**^b^ | **Indel *MUC13***^c^ |
| --- | --- | --- | --- | --- | --- | --- | --- | --- | --- | --- | --- | --- | --- |
| V1 | 1 | S1 | B1 | hybrid | SS | BB | V69 | 3 | S3 | B1 | hybrid | SR | BB |
| V2 | 1 | S1 | B1 | hybrid | SS | BB | V70 | 4 | S4 | B3 | hybrid | RR | AB |
| V3 | 1 | S1 | B1 | hybrid | SS | AB | V71 | 4 | S4 | B3 | hybrid | RR | AB |
| V4 | 1 | S1 | B1 | hybrid | SR | BB | V72 | 4 | S4 | B3 | hybrid | RR | AB |
| V5 | 1 | S1 | B1 | hybrid | SS | AB | V73 | 4 | S4 | B3 | hybrid | RR | AB |
| V6 | 1 | S1 | B1 | hybrid | RR | BB | V74 | 4 | S4 | B3 | hybrid | RR | AB |
| V7 | 2 | S2 | B2 | hybrid | RR | BB | V75 | 4 | S4 | B3 | hybrid | RR | AB |
| V8 | 2 | S2 | B2 | hybrid | SS | BB | V76 | 5 | S5 | B4 | hybrid | RR | BB |
| V9 | 2 | S2 | B2 | hybrid | SR | BB | V77 | 5 | S5 | B4 | hybrid | RR | BB |
| V10 | 2 | S2 | B2 | hybrid | SS | BB | V78 | 6 | S6 | B5 | hybrid | RR | AB |
| V11 | 3 | S3 | B1 | hybrid | SR | BB | V79 | 6 | S6 | B5 | hybrid | RR | AB |
| V12 | 5 | S5 | B4 | hybrid | SR | BB | V80 | 7 | S7 | B6 | hybrid | RR | AB |
| V13 | 5 | S5 | B4 | hybrid | SR | BB | V81 | 8 | S8 | B7 | LW x P | RR | AA |
| V14 | 6 | S6 | B5 | hybrid | SR | BB | V82 | 11 | S9 | B8 | LW x BL | RR | AB |
| V15 | 7 | S7 | B6 | hybrid | SR | BB | V83 | 12 | S10 | B9 | LW | RR | AA |
| V16 | 8 | S8 | B7 | LW x P | SS | BB | V84 | 13 | S11 | B8 | LW x BL | RR | BB |
| V17 | 8 | S8 | B7 | LW x P | SR | BB | V85 | 14 | S12 | B8 | LW x BL | RR | BB |
| V18 | 8 | S8 | B7 | LW x P | SS | BB | V86 | 15 | S13 | B10 | LW x P | RR | AA |
| V19 | 8 | S8 | B7 | LW x P | SS | BB | V87 | 17 | S14 | B8 | LW x BL | RR | AB |
| V20 | 11 | S9 | B8 | LW x BL | SR | BB | V88 | 18 | S15 | B11 | LW x P | RR | AB |
| V21 | 12 | S10 | B9 | LW | SR | AB | V89 | 21 | S16 | B12 | LW x BL | RR | AB |
| V22 | 13 | S11 | B8 | LW x BL | SS | BB | V90 | 22 | S17 | B13 | hybrid | RR | AB |
| V23 | 14 | S12 | B8 | LW x BL | SS | BB | V91 | 23 | S18 | B13 | hybrid | RR | BB |
| V24 | 17 | S14 | B8 | LW x BL | SR | AB | V92 | 24 | S19 | B14 | hybrid | RR | BB |
| V25 | 17 | S14 | B8 | LW x BL | SS | BB | V93 | 25 | S20 | B15 | hybrid | RR | BB |
| V26 | 18 | S15 | B10 | LW x P | SR | AB | V94 | 29 | S24 | B12 | LW | RR | AA |
| V27 | 24 | S19 | B14 | hybrid | RR | BB | V95 | 30 | S25 | B16 | LW x P | RR | AA |
| V28 | 25 | S20 | B15 | hybrid | RR | BB | V96 | 31 | S26 | B17 | hybrid | RR | AB |
| V29 | 25 | S20 | B15 | hybrid | SR | BB | V97 | 34 | S29 | B17 | hybrid | RR | AB |
| V30 | 25 | S20 | B15 | hybrid | RR | BB | V98 | 34 | S29 | B17 | hybrid | RR | AB |
| V31 | 25 | S20 | B15 | hybrid | RR | BB | V99 | 35 | S30 | B17 | hybrid | RR | AB |
| V32 | 26 | S21 | B15 | hybrid | SR | BB | V100 | 37 | S32 | B19 | BL | RR | AB |
| V33 | 26 | S21 | B15 | hybrid | SR | BB | V101 | 38 | S33 | B20 | LW | RR | AA |
| V34 | 27 | S22 | B15 | hybrid | SS | BB | V102 | 39 | S34 | B18 | BL | RR | AB |
| V35 | 27 | S22 | B15 | hybrid | SR | BB | V103 | 40 | S35 | B21 | LW | RR | AA |
| V36 | 27 | S22 | B15 | hybrid | SS | BB | V104 | 40 | S35 | B21 | LW | RR | AA |
| V37 | 28 | S23 | B15 | hybrid | SR | BB | V105 | 40 | S35 | B21 | LW | RR | AA |
| V38 | 28 | S23 | B15 | hybrid | SR | BB | V106 | 40 | S35 | B21 | LW | RR | AA |
| V39 | 29 | S24 | B12 | LW | SR | AB | V107 | 41 | S36 | B22 | LW | RR | AA |
| V40 | 29 | S24 | B12 | LW | SS | BB | V108 | 41 | S36 | B22 | LW | RR | AB |
| V41 | 30 | S25 | B16 | LW x P | SR | AB | V109 | 41 | S36 | B22 | LW | SR | AA |
| V42 | 30 | S25 | B16 | LW x P | SS | BB | V110 | 41 | S36 | B22 | LW | SR | AA |
| V43 | 32 | S27 | B17 | hybrid | SR | BB | V111 | 42 | S37 | B22 | LW | RR | AB |
| V44 | 32 | S27 | B17 | hybrid | RR | BB | V112 | 43 | S38 | B22 | LW | RR | AB |
| V45 | 32 | S27 | B17 | hybrid | SS | BB | V113 | 43 | S38 | B22 | LW | RR | AB |
| V46 | 33 | S28 | B17 | hybrid | SR | BB | V114 | 43 | S38 | B22 | LW | RR | AB |
| V47 | 33 | S28 | B17 | hybrid | RR | AB | V115 | 43 | S38 | B22 | LW | RR | AA |
| V48 | 34 | S29 | B17 | hybrid | SR | BB | V116 | 45 | S39 | B22 | LW | RR | AA |
| V49 | 35 | S30 | B17 | hybrid | SR | BB | V117 | 46 | S40 | B23 | LW | RR | AB |
| V50 | 35 | S30 | B17 | hybrid | RR | AB | V118 | 46 | S40 | B23 | LW | RR | AA |
| V51 | 36 | S31 | B18 | LW x BL | SS | BB | V119 | 46 | S40 | B23 | LW | RR | AA |
| V52 | 36 | S31 | B18 | LW x BL | SR | AB | V120 | 48 | S42 | B23 | LW x BL | SR | AB |
| V53 | 36 | S31 | B18 | LW x BL | SR | AB |  |  |  |  |  |  |  |
| V54 | 37 | S32 | B19 | BL | SR | AB |  |  |  |  |  |  |  |
| V55 | 37 | S32 | B19 | BL | RR | BB |  |  |  |  |  |  |  |
| V56 | 38 | S33 | B20 | LW | SR | AB |  |  |  |  |  |  |  |
| V57 | 38 | S33 | B20 | LW | SR | AB |  |  |  |  |  |  |  |
| V58 | 39 | S34 | B18 | BL | SR | BB |  |  |  |  |  |  |  |
| V59 | 39 | S34 | B18 | BL | SR | BB |  |  |  |  |  |  |  |
| V60 | 42 | S37 | B22 | BL | SR | BB |  |  |  |  |  |  |  |
| V61 | 42 | S37 | B22 | BL | SR | AB |  |  |  |  |  |  |  |
| V62 | 42 | S37 | B22 | BL | SR | AB |  |  |  |  |  |  |  |
| V63 | 45 | S39 | B22 | BL | SR | AB |  |  |  |  |  |  |  |
| V64 | 45 | S39 | B22 | BL | SR | BB |  |  |  |  |  |  |  |
| V65 | 45 | S39 | B22 | BL | SR | BB |  |  |  |  |  |  |  |
| V66 | 47 | S41 | B23 | LW x BL | SR | AB |  |  |  |  |  |  |  |
| V67 | 48 | S42 | B23 | LW x BL | SR | BB |  |  |  |  |  |  |  |
| V68 | 49 | S43 | B23 | LW x BL | SR | BB |  |  |  |  |  |  |  |
