# Supplementary material for: Refined Candidate Region for F4ab/ac Enterotoxigenic Escherichia coli Susceptibility Situated Proximal to MUC13 in Pigs
Source: PLoS One. 2014 Aug 19;9(8):e105013. doi: 10.1371/journal.pone.0105013 (PMC4138166; doi:10.1371/journal.pone.0105013)
Supplement: Table S2 — Significant SNPs for F4ab/ac ETEC susceptibility. aDerived from porcine GenomeBuild 10.2; bThe nearest annotated porcine gene to the significant SNP based on the porcine GenomeBuild 10.2. Numbers in parentheses indicate distance in base pairs (bp); cGenomic control corrected significance value. This is based on a simple estimation of the inflation factor based on median chi-square statistic (genomic inflation factor λ is 1.26636) [25]. (DOCX) [file pone.0105013.s002.docx]

Table S2.

| Chr. | Position (bp)^a^ | SNP name | Nearest gene^b^ | Genomic control corrected *P*-values^c^ | Bonferroni corrected *P*-values | max(T) empirical *P***-**values |
| --- | --- | --- | --- | --- | --- | --- |
| 13 | 144,946,742 | ASGA0089965 | *MUC13* (45,916 bp) | 1.29E-20 | 6.19E-21 | 1.00E-04 |
| 13 | 144,981,309 | ASGA0091537 | *MUC13* (11,349 bp) | 1.29E-20 | 6.19E-21 | 1.00E-04 |
| 13 | 145,009,805 | ALGA0106330 | *MUC13* (within) | 1.14E-16 | 5.97E-16 | 1.00E-04 |
| 13 | 144,299,267 | ASGA0095873 | *LMLN* (within) | 9.24E-13 | 5.11E-11 | 1.00E-04 |
| 13 | 143,820,612 | MUC4 | *MUC4* (within) | 2.87E-13 | 1.17E-11 | 1.00E-04 |
| 13 | 144,832,256 | ALGA0072075 | *HEG1* (31,668 bp) | 3.17E-12 | 2.42E-10 | 1.00E-04 |
| 13 | 144,733,031 | ASGA0058925 | *SLC12A8* (within) | 8.48E-12 | 8.37E-10 | 1.00E-04 |
| 13 | 145,772,058 | MARC0088848 | *HEG1* (within) | 1.90E-11 | 2.31E-09 | 1.00E-04 |
| 13 | 145,732,401 | ASGA0058958 | *SLC12A8* (within) | 2.80E-11 | 3.78E-09 | 1.00E-04 |
| 13 | 145,671,763 | ALGA0072105 | *SLC12A8* (within) | 3.64E-11 | 5.26E-09 | 1.00E-04 |
| 13 | 143,656,188 | ASGA0058885 | *SLC12A8* (within) | 5.09E-11 | 8.03E-09 | 1.00E-04 |
| 13 | 145,398,474 | MARC0045442 | *UMPS* (243582 bp) | 1.51E-10 | 3.16E-08 | 1.00E-04 |
| 13 | 144,197,577 | ALGA0072072 | *LMLN* (within) | 7.37E-10 | 2.33E-07 | 1.00E-04 |
| 13 | 144,167,475 | ALGA0072065 | *IQCG* (within) | 7.37E-10 | 2.33E-07 | 1.00E-04 |
| 13 | 144,145,817 | ALGA0072067 | *IQCG* (within) | 7.37E-10 | 2.33E-07 | 1.00E-04 |
| 13 | 144,946,317 | ALGA0122555 | *MUC13*(46,341 bp) | 1.72E-09 | 6.77E-07 | 1.00E-04 |
| 13 | 144,993,222-144,993,289 | Indel MUC13 | *MUC13* (within) | 1.72E-09 | 6.77E-07 | 1.00E-04 |
| 0 | 0 | ALGA0122702 | NA | 2.49E-09 | 1.08E-06 | 1.00E-04 |
| 13 | 144,094,647 | MARC0089106 | *IQCG* (40,326 bp) | 3.48E-09 | 1.65E-06 | 1.00E-04 |
| 13 | 144,126,389 | MARC0043596 | *IQCG* (8,584 bp) | 3.48E-09 | 1.65E-06 | 1.00E-04 |
| 13 | 143,866,440 | ALGA0072062 | *MUC20* (within) | 8.20E-09 | 4.85E-06 | 1.00E-04 |
| 13 | 147,328,480 | ALGA0072162 | *SEMA5B* (16,459 bp) | 8.30E-09 | 4.92E-06 | 1.00E-04 |
| 13 | 147,911,293 | ISU10000469 | *CASR* (within) | 5.42E-08 | 5.23E-05 | 0.0004 |
| 13 | 146,433,577 | H3GA0037388 | *PTPLB* (174,712) | 5.64E-08 | 5.50E-05 | 0.0004 |
| 13 | 142,313,068 | ASGA0058867 | *APOD* (218,113) | 6.64E-08 | 6.75E-05 | 0.0005999 |
| 13 | 145,414,240 | DIAS0000584 | *UMPS* (259,348 bp) | 7.50E-08 | 7.87E-05 | 0.0006999 |
| 13 | 143,624,457 | M1GA0017682 | *TNK2* (22,741 bp) | 1.03E-07 | 0.00012 | 0.0011 |
| 13 | 143,618,378 | MARC0012378 | *TNK2* (28,820 bp) | 1.03E-07 | 0.00012 | 0.0011 |
| 13 | 148,020,127 | MARC0031951 | *CASR* (85,108 bp) | 2.38E-07 | 0.00034 | 0.0013 |
| 13 | 146,824,849 | H3GA0037402 | *PDIA5* (18,255 bp) | 2.49E-07 | 0.00036 | 0.0013 |
| 13 | 143,638,483 | MARC0093203 | *TNK2* (8,715 bp) | 3.11E-07 | 0.00047 | 0.0013 |
| 13 | 143,825,858 | ASGA0058906 | *MUC4* (within) | 5.75E-07 | 0.00102 | 0.0025 |
| 13 | 147,415,740 | MARC0032449 | *HASPBAP1* (within) | 7.35E-07 | 0.00139 | 0.0031 |
| 13 | 147,295,491 | DIAS0001133 | *SEMA5B* (within) | 7.55E-07 | 0.00144 | 0.0034 |
| 13 | 145,473,321 | H3GA0037371 | *UMPS* (318,429) | 7.88E-07 | 0.00152 | 0.0034 |
| 13 | 146,909,376 | M1GA0017695 | *PDIA5* (within) | 9.70E-07 | 0.00197 | 0.0043 |
| 13 | 145,096,895 | ALGA0072090 | *ITGB5* (within) | 1.50E-06 | 0.00339 | 0.005999 |
| 13 | 143,307,737 | H3GA0037321 | *SLC51A* (119 bp) | 2.23E-06 | 0.00559 | 0.007899 |
| 0 | 0 | M1GA0027009 | NA | 3.61E-06 | 0.01024 | 0.013 |
| 13 | 144,611,608 | MARC0067282 | *SLC12A8* (19,579 bp) | 5.85E-06 | 0.0188 | 0.0201 |
| 13 | 144,488,410 | MARC0099692 | *ZBP-89* (28,751 bp) | 5.85E1-06 | 0.0188 | 0.0201 |
| 13 | 147,927,896 | ASGA0059010 | *CASR* (within) | 6.62E-06 | 0.02197 | 0.0232 |
| 13 | 144,781,809 | ASGA0058923 | *HEG1* (within) | 7.22E-06 | 0.0245 | 0.0247 |
| 13 | 147,536,783 | MARC0105487 | *PARP15* (5097 bp) | 1.22E-05 | 0.04727 | 0.0374 |
