# Supplementary material for: Refined Candidate Region for F4ab/ac Enterotoxigenic Escherichia coli Susceptibility Situated Proximal to MUC13 in Pigs
Source: PLoS One. 2014 Aug 19;9(8):e105013. doi: 10.1371/journal.pone.0105013 (PMC4138166; doi:10.1371/journal.pone.0105013)
Supplement: Protocol S1 — Protocol of the MUC4 TaqMan assay using the iCycler iQ Real-Time PCR Detection System Software version 3.0a (Bio-Rad Laboratories, USA). (A) Oligonucleotide sequences of primers and probes with their specifications (MUC4: DQ848681). Fluorescent labels and quenchers are in italic; SNPs are underlined. (B) PCR-mix (10 µl) used in the MUC4 TaqMan assay by Real-Time PCR. (C) PCR program used in the MUC4 TaqMan assay by Real-Time PCR. (DOCX) [file pone.0105013.s003.docx]

Protocol S1.

A.

| Primer-probe | Sequence (5’→3’) | Tm (◦C) |
| --- | --- | --- |
| Forward primer | TTACAACAACCCCATGAAG | 56.51 |
| Reverse primer | GGTGAGAGGTTAATTTCCAG | 56.87 |
| G-probe (susceptible allele) | /*5TEX615*/ACCCATTCTAGAGATACAGAAAC/*3BHQ 2*/ | 58.88 |
| C-probe (resistant allele) | /*56-FAM*/ACCCATTCTACAGATACAGAAAC/*3IABkFQ*/ | 58.36 |

B.

| 2×PerfeCTa^TM^ qPCR FastMIX^TM^, UNG (Quanta Biosciences) |
| --- |
| 10 μM G-probe (susceptible allele) |
| 10 μM C-probe (resistant allele) |
| 10 μM Forward primer |
| 10 μM Reverse primer |
| DNA |

C.

| 3 min | 95°C |  |  |
| --- | --- | --- | --- |
| 20 s | 95°C | 40 cycles |  |
| 40 s | 56°C |  | detection |
